# Antiproliferative Factor-Induced Changes in Phosphorylation and Palmitoylation of Cytoskeleton-Associated Protein-4 Regulate Its Nuclear Translocation and DNA Binding

**DOI:** 10.1155/2012/150918

**Published:** 2012-03-20

**Authors:** David A. Zacharias, Matthew Mullen, Sonia Lobo Planey

**Affiliations:** ^1^Whitney Laboratory, Department of Neuroscience, University of Florida, St. Augustine, FL 32080, USA; ^2^Department of Basic Sciences, The Commonwealth Medical College, Scranton, PA 18509, USA

## Abstract

Cytoskeleton-associated protein 4 (CKAP4) is a reversibly palmitoylated and phosphorylated transmembrane protein that functions as a high-affinity receptor for antiproliferative factor (APF)—a sialoglycopeptide secreted from bladder epithelial cells of patients with interstitial cystitis (IC). Palmitoylation of CKAP4 by the palmitoyl acyltransferase, DHHC2, is required for its cell surface localization and subsequent APF signal transduction; however, the mechanism for APF signal transduction by CKAP4 is unknown. In this paper, we demonstrate that APF treatment induces serine phosphorylation of residues S3, S17, and S19 of CKAP4 and nuclear translocation of CKAP4. Additionally, we demonstrate that CKAP4 binds gDNA in a phosphorylation-dependent manner in response to APF treatment, and that a phosphomimicking, constitutively nonpalmitoylated form of CKAP4 localizes to the nucleus, binds DNA, and mimics the inhibitory effects of APF on cellular proliferation. These results reveal a novel role for CKAP4 as a downstream effecter for APF signal transduction.

## 1. Introduction

Cytoskeleton-associated protein 4 (CKAP4; also known as CLIMP-63, ERGIC-63, and p63) is a 63 kDa, reversibly palmitoylated and phosphorylated, type II transmembrane (TM) protein, originally identified as a resident of the endoplasmic reticulum/Golgi intermediate complex (ERGIC) [1–5]. The first report describing CKAP4 [1] (referring to it as “p62”) demonstrated that its palmitoylation peaked during mitosis and suggested that palmitoylation may be an important regulator of vesicular transport between various membranous compartments. Soon thereafter, Schweizer and colleagues cloned CKAP4 and identified membrane-proximal cysteine 100 (C100) as the site for palmitoylation [4]. More recently, DHHC2 was identified as the palmitoyl acyltransferase (PAT) that palmitoylates CKAP4 at C100 [6].

**Figure 1:**
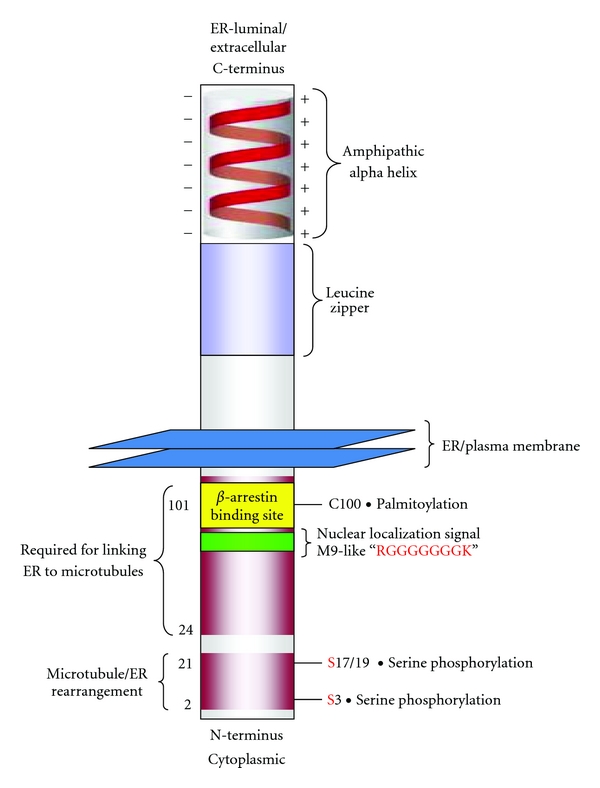
*CKAP4 domains*. CKAP4 is a 63 kDa, oligomeric, type II, single-pass TM domain protein that is palmitoylated and phosphorylated. The luminal/extracellular domain contains an amphipathic alpha helical region (506-KVQEQVHTLLSQDQA QAARLPPQDFLDRLSSLDNLKASVSQVEADLKMLRTAVDSLVAYSV KIETNENNLESAKGLLDDLRNDLDRLFVKVEKIHEKV-602) that is important for oligomerization and a leucine zipper (468-**L**ASTVRS**L**GETQLV**L**YGDVEE**L**KRSVGE**L**PSTVES**L**-504). Together, these regions are homologous to the DNA-binding domain of bZIP transcription factors. The cytoplasmic, N-terminus contains a cysteine residue adjacent to the TM domain at position 100 that is palmitoylated (a modification that is important for trafficking from the ER to the PM), and three serine residues (S3, S17, and S19) that are required for phosphorylation-dependent binding of CKAP4 to the microtubule cytoskeleton. Two regions in the cytoplasmic N-terminus are required for binding to and bundling microtubules, thereby maintaining the connection between the ER and the cytoskeleton [3]. There is also a PQ protein-protein interaction domain (49-PHPQQHPQQHPQNQ-63) and a putative glycine-rich nuclear localization signal sequence (65-GKGGHRGGGGGGGK-79).

CKAP4 is localized prominently to the endoplasmic reticulum (ER). One major function of CKAP4 is to anchor rough ER to microtubules, organizing the overall structure of ER with respect to the microtubule network [3–5, 7, 8]. The binding of CKAP4 to microtubules is regulated by phosphorylation of three critical serine residues (S3, S17, and S19) located in its cytosolic, N-terminal domain (Figure 1) [2]. CKAP4 is unique among microtubule binding proteins in at least one respect: it is a TM protein. However, it is similar to many other microtubule-binding proteins in that phosphorylation blocks its ability to bind microtubules [9]. Overexpression of a mutant version of CKAP4 that mimics phosphorylation of three serine residues (S3E, S17E, and S19E) within the microtubule binding domain results in a restructuring or “collapse” of the ER around the nucleus without any observable effect on the microtubule network. Similar effects on the ER structure occurred when a deletion mutant of CKAP4 lacking the same serine residues was overexpressed in cells. Conversely, overexpression of a full-length, phosphorylation-incompetent (S3A, S17A, and S19A) mutant of CKAP4 colocalized with and was able to bundle microtubules similar to wild-type CKAP4 [7].

CKAP4 is also expressed on the plasma membrane (PM). Palmitoylation by DHHC2 is required for expression of CKAP4 on the cell surface [6], where it functions as a high-affinity receptor for antiproliferative factor (APF) [10]. APF is a lipophilic, nine-residue sialoglycopeptide with an amino acid sequence identical to the putative 6th TM domain of Frizzled 8 [11]. APF is present in the urine of patients with interstitial cystitis (IC), a chronic, painful bladder disease with a poorly understood etiology [12]. Exposing cells to APF *in vitro* dramatically alters gene expression and blocks proliferation of normal bladder epithelial cells and cancer cell lines including bladder (T24) and cervical (HeLa) adenocarcinoma, mimicking critical aspects of the pathology of the bladder epithelium in IC patients [6, 11, 13, 14]. The IC_50_ of synthetic and purified APF in proliferation assays is ~1 nM [14, 15], indicating that the affinity of APF for CKAP4 is high.

Previously, we observed an increase in the nuclear abundance of CKAP4 in HeLa cells following exposure to APF [6]. Importantly, this APF-induced change in CKAP4 localization was dependent on palmitoylation by DHHC2. Concurrent with the increased nuclear abundance of CKAP4, the expression level of several genes, (i.e., vimentin, zonula occludens-1, and E-cadherin) changed significantly in HeLa and normal bladder epithelial cells following APF exposure [6, 10, 13, 16]. These genes are among thirteen others shown to have significantly altered expression in bladder tissue from IC patients and known to be involved in the regulation of proliferation, cell adhesion, and tumorigenesis [17–19]. The redistribution of CKAP4 to the nucleus and the concurrent changes in gene expression suggest that APF induces specific changes in the palmitoylation and/or phosphorylation state of CKAP4, and that these changes affect its subcellular distribution and function within the cell. Consequently, we generated CKAP4 mutants that mimic constitutive depalmitoylation and various states of serine phosphorylation to determine their effect on the subcellular distribution of CKAP4 in response to APF.

Our results show that APF increases serine phosphorylation of CKAP4, and that phosphorylation of residues S3, S17, and S19 are required for its nuclear translocation; moreover, a phosphomimicking, constitutively nonpalmitoylated form of CKAP4 localizes to the nucleus, binds DNA, and mimics the inhibitory effects of APF on cellular proliferation. These results reveal a novel role for CKAP4 in APF-dependent signaling from the plasma membrane to the nucleus and suggest that CKAP4 may regulate transcription by binding directly to DNA.

## 2. Experimental Procedures

### 2.1. DNA Constructs

A vector construct containing wild-type CKAP4 (WT CKAP4) fused in-frame to the N-terminus of the V5 and 6xHis epitope tags in the mammalian expression vector pcDNA3.1V5His6-TOPO (Invitrogen, Carlsbad, CA) was generated by PCR using CKAP4-specific primers and cDNA from HeLa cells. A palmitoylation-incompetent form of CKAP4 (CKAP4C100S) was created by site-directed mutagenesis of the wild-type vector construct (Stratagene, La Jolla, CA) to change the cysteine at position 100 to serine. A series of CKAP4 mutants that mimic depalmitoylation and/or constitutive dephosphorylation and phosphorylation were constructed by site-directed mutagenesis of the WT CKAP4 or CKAP4C100S vector constructs as described in Table 1. cDNA encoding the extracellular domain of CKAP4 (C-terminal residues 126–501) was generated by PCR and cloned in-frame with the His6 epitope tag of pRSET-B (Invitrogen) at the 5′ end. The accuracy of all constructs was verified by DNA sequencing.

### 2.2. Cell Culture and Transfections

HeLa (ATCC #CCL-2; American Type Culture Collection, Manassas, VA) cells were maintained in Dulbecco's modified Eagle's medium (DMEM) containing 10% fetal bovine serum (FBS), 100 U/mL penicillin, and 100 *μ*g/mL streptomycin and 1 *μ*g/mL fungizone (all from Invitrogen). Cells were transfected using FuGENE6 reagent (Roche, Basel, Switzerland) according to the manufacturer's instructions.

### 2.3. Immunocytochemistry

HeLa cells expressing WT CKAP4 or CKAP4 mutants that mimic depalmitoylation and/or constitutive dephosphorylation and phosphorylation (see Table 1) were seeded at a density of 2 × 10^4^ cells/well in 8-well LabTek chamber slides (Nalge Nunc, Rochester, NY) and grown to semiconfluence in DMEM medium containing 10% FBS, 100 U/mL penicillin, 100 *μ*g/mL streptomycin, 1 *μ*g/mL fungizone, and 0.4 mg/mL G418 (all from Invitrogen). Cells were fixed for 20 minutes with 3% paraformaldehyde in phosphate-buffered saline (PBS), permeabilized with 0.1% Triton X-100 in PBS, and blocked in PBS/5% normal goat serum (NGS). The following primary antibodies were used: mouse mAb G1/296 against CKAP4 (“anti-CLIMP-63”, diluted 1 : 100, Alexis Biochemicals, San Diego, CA), rabbit pAb against tubulin (diluted 1 : 100, Abcam, Cambridge, MA), rabbit pAb against fibrillarin (diluted 1 : 100, Abcam), and fluorescein isothiocyanate- (FITC-) conjugated mouse mAb against the V5 epitope (diluted 1 : 500, Invitrogen). Secondary antibodies were FITC-labeled goat anti-rabbit or goat anti-mouse (diluted 1 : 1000, Invitrogen) and tetramethyl rhodamine isothiocyanate- (TRITC-) labeled goat anti-mouse (diluted 1 : 1000, Jackson ImmunoResearch Laboratories, West Grove, PA). Slides were mounted in SlowFade Antifade reagent (Invitrogen) and imaged using a Nikon TE2000 epifluorescence microscope or Leica SP5II confocal.

### 2.4. Nuclear and Nucleolar Protein Isolation

HeLa cells were serum-starved overnight and then treated with 20 nM synthetic APF (Peptides International) for 24 hours. Nuclear extracts were generated using the NE-PER kit (ThermoFisher Scientific, Waltham, MA) according to the manufacturer's instructions. Nucleoli were isolated using a variation on the method used by Busch and coworkers in 1963 [20] as described by the Lamond lab (http://www.lamondlab.com/f7protocols.htm). Briefly, APF-treated or untreated HeLa cells were washed with cold PBS (pH 7.4), trypsinized, and collected by centrifugation at 500 ×g for 5 min. The cell pellets were resuspended in 15 volumes of a hypotonic buffer (10 mM Tris-HCl, pH 7.4, 10 mM NaCl, and 1 mM MgCl_2_), incubated on ice for 30 min, and then lysed by adding Nonidet P-40 to a final concentration of 0.3%. The samples were homogenized 10 times using a glass dounce homogenizer (0.4-mm clearance; Kimberly/Kontes, Owens, IL) while keeping the homogenizer on ice. Nuclei were collected by centrifugation at 218 ×g for 5 min and resuspended in 10 volumes of 0.25 M sucrose containing 10 mM MgCl_2_. Nuclei were then purified by centrifugation at 1430 ×g for 5 min through a 0.88 M sucrose cushion containing 0.05 mM MgCl_2_. Purified nuclei were resuspended in 10 volumes of 0.34 M sucrose containing 0.05 mM MgCl_2_ and sonicated on ice for six, 10-second bursts with 10-second intervals between each burst. Nucleoli were then purified from the resulting homogenate by centrifugation at 3000 ×g for 10 min through a 0.88 M sucrose cushion containing 0.05 mM MgCl_2_. Purified nucleoli were resuspended in 0.34 M sucrose containing 0.05 mM MgCl_2_ for further analyses.

### 2.5. Immunoprecipitation

Lysates (500 *μ*g) from serum-starved HeLa cells treated with 20 nM APF or serum-starved controls were immunoprecipitated with an mAb antibody against CKAP4 (G1/296; 1 *μ*g mAb/100 *μ*g total protein lysate; Alexis Biochemicals) overnight at 4°C. Samples were then bound to Protein A, washed (4X with RIPA/Empigen buffer), eluted (4X LDS sample buffer; Invitrogen), boiled at 95°C for 5 min and resolved on a 4–12% Bis-Tris gel and transferred to a nitrocellulose membrane under reducing conditions. Western Blot analysis detected phosphoserine on CKAP4 using primary mouse mAb, *α*-phosphoserine antibody (Invitrogen; diluted 1 : 1000) and secondary, HRP-conjugated goat anti-mouse (diluted 1 : 20,000, Jackson ImmunoResearch Laboratories). The signal was detected on film by ECL (ThermoFisher Scientific). The membrane was stripped and reprobed with a mouse mAb G1/296 against CKAP4 (Alexis Biochemicals) to normalize the phosphoserine signal to the amount of immunoprecipitated CKAP4. Densitometric analysis of the immunoreactive bands was performed using ImageJ, and the ratios of phosphorylated to nonphosphorylated CKAP4 were determined.

### 2.6. Western Blot Analysis

Cells were lysed in ice-cold RIPA buffer containing protease inhibitors (ThermoFisher Scientific), sonicated, and centrifuged for 15 minutes at 4°C. The supernatant protein concentration was measured using the Micro BCA protein assay kit (ThermoFisher Scientific). Proteins were separated by electrophoresis using 4–12% NuPAGE Novex Bis-Tris polyacrylamide gels in MOPS running buffer (Invitrogen) and then transferred to nitrocellulose. Membranes were blocked for 2 hours at room temperature in TBST buffer (Tris-buffered saline, pH 7.4, with 0.1% Tween 20) containing 5% nonfat milk and incubated with an mAb antibody against CKAP4 (G1/296; diluted 1 : 1000; Alexis Biochemicals) overnight at 4°C. The membrane was subsequently washed with TBST, incubated for 1 hour at room temperature in HRP-conjugated goat, anti-mouse (diluted 1 : 20000; ThermoFisher Scientific) secondary antibody and developed by enhanced chemiluminescence (ThermoFisher Scientific). To assess equal loading of protein, the membranes were stripped and reprobed for *β*-tubulin (diluted 1 : 1000; Abcam) and also for fibrillarin (diluted 1 : 1000; Abcam) to ensure the purity of the nuclear fraction. The membranes were exposed to film (BioMax AR, Kodak, Rochester, NY) and the resulting images scanned at 300 dpi. The protein bands of interest were quantified using ImageJ and the integrated signal densities normalized to *β*-tubulin (cytosol) or fibrillarin (nuclear) and subsequently expressed in terms of the fractional abundance relative to untreated control cells.

### 2.7.  ^32^P Metabolic Labeling

HeLa cells grown to ~80% confluence in 10 cm dishes were serum starved for three hours and then metabolically labeled with 150 *μ*Ci *γ* ^32^P-ATP for one hour. The cells were then exposed to APF (20 nM; 24 hours) or left in serum-free medium (control) for 24 hours. The cells were then harvested, washed three times in ice-cold PBS, and the nuclear fraction was isolated using the NE-PER kit (ThermoFisher Scientific). Equal quantities of each fraction were separated by SDS-PAGE and transferred to nitrocellulose for Western Blot analysis using a CKAP4 mAb (G1/296; diluted 1 : 500; Alexis Biochemicals) as described above. Following the Western Blot, the membrane was rinsed in 1% H_2_O_2_ for one minute to eliminate the chemiluminescent signal and then wrapped in plastic wrap and exposed to film for 12 hours to detect the phosphorylated proteins.

### 2.8. Cell Surface Biotinylation

Sulfo-NHS-biotin (ThermoFisher Scientific) was dissolved in serum-free DMEM to a final concentration of 0.5 mg/mL. HeLa cells were grown in serum-free medium for 36 hours (10 cm dishes at ~80% confluence), washed three times in PBS, and the medium replaced with serum-free DMEM/Biotin. The labeling reaction proceeded at 4°C for 60 minutes. Next, the cells were washed three times in PBS/100 mM gylcine to quench unreacted biotin. Serum-free DMEM containing APF (20 nM) or without APF (control) was added back to the cells, which were then incubated at 37°C for four hours. Cells were harvested by trypsinization, washed three times in PBS, and the nuclear protein fraction was isolated using the NE-PER kit (ThermoFisher Scientific) according to the manufacturer's protocol. Equal quantities of protein from each nuclear fraction were separated by SDS-PAGE and then blotted to nitrocellulose. The membrane was blocked overnight at 4°C in TBST buffer containing 10% milk and subsequently washed three times in TBS, once in TBST and once with PBS. The membrane was then incubated two hours at room temperature in PBS/1% BSA containing Streptavidin HRP (ThermoFisher Scientific) at a concentration of 1 : 500 dilution (0.02 *μ*g/mL). The membrane was then washed three times (10 minutes each) at 4°C in TBS and once in TBST. Biotinylated proteins on the membrane were detected using ECL (ThermoFisher Scientific; Super Signal West Pico). The same membrane was subsequently Western Blotted simultaneously with antibodies to CKAP4 (G1/296; diluted 1 : 1000; Alexis Biochemicals) and fibrillarin (diluted 1 : 100, Abcam). The signal from the biotinylated CKAP4 in the nuclear fraction was normalized to the nuclear abundance of CKAP4 as determined by Western Blot and the total amount of nuclear protein loaded into each lane as was determined by the signal from fibrillarin.

### 2.9. Genomic DNA Affinity Chromatography (GDAC)

HeLa cells treated with APF (or control) or expressing CKAP4C100S/SΔE (for 24–48 hours) were scraped into gDNA binding buffer (20 mM Tris [pH 7.5], 100 mM KCl, 10% Glycerol, 1 mM EDTA, 1 mM DTT, 1 mg/mL BSA, 0.1% SDS) with 1X phosphatase inhibitor and 1X phosphatase inhibitor cocktail 1 (both from ThermoFisher Scientific). Proteins were extracted for 2 hours on ice and then the insoluble fraction was pelleted by centrifugation at 15,000 ×g for 10 minutes at 4°C. Next, 15–100 mg of gDNA cellulose or cellulose alone (control; both from Sigma) was added to the cleared supernatant and mixed by constant inversion on a rotating wheel at 4°C for two hours to overnight. To capture the bacterially expressed C-terminus of CKAP4, 0.4–40 *μ*g of purified protein (as determined by the Micro BCA assay, ThermoFisher Scientific) was added to 15–50 mg gDNA-cellulose in 500 *μ*L of gDNA-binding buffer and mixed as above at 4°C. After proteins were allowed to bind, the gDNA-cellulose or cellulose alone was pelleted by centrifugation and washed with PBS three times. This wash protocol was sufficient to remove nonspecifically bound proteins and nonphosphorylated CKAP4 from the cellulose alone and from the gDNA-cellulose. After the final wash, SDS-PAGE sample buffer was added to 10 *μ*L of the supernatant previously removed from the gDNA pellet, the cellulose, or gDNA-cellulose pellet. The samples were heated for 5 minutes at 95°C, separated by SDS-PAGE, transferred to nitrocellulose, and probed with *α*-V5 antibody (mAb; 1: 5000; Invitrogen) followed by goat-*α*-mouse-HRP (1 : 10,000, ThermoFisher Scientific) to detect the transiently expressed CKAPC100S/SΔE or *α*-CKAP4 (mAb G1/296; 1 : 1000; Alexis Biochemicals) followed by goat-*α*-mouse-HRP (1 : 10,000, ThermoFisher Scientific) to detect endogenous and/or transiently expressed CKAP4. The signal was detected on film using chemiluminescence (SuperSignal West Pico, ThermoFisher Scientific).

## 3. Results

### 3.1. CKAP4 Translocates from the Plasma Membrane to the Nucleus in Response to APF

**Figure 2:**
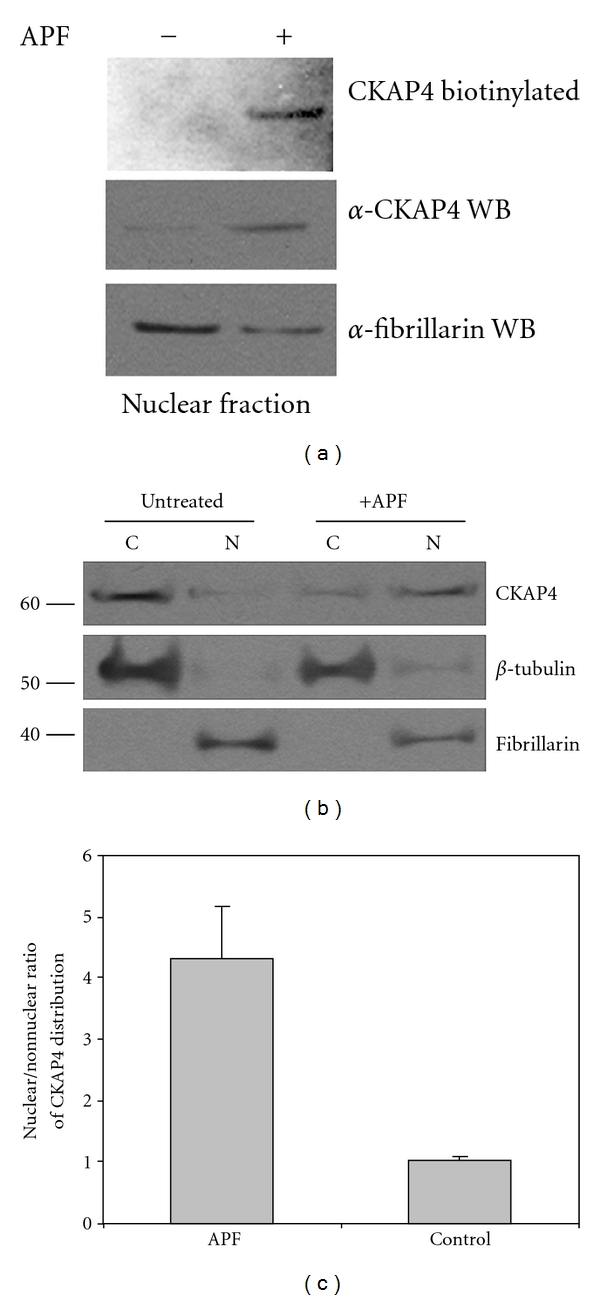
*Surface-labeled CKAP4 translocates from the plasma membrane into the nucleus following APF exposure*. (a) HeLa cell-surface proteins were labeled with Sulfo NHS-biotin as described in Section 2. Following exposure to 20 nM APF for 24 hours (or no treatment), the cells were harvested and the nuclear protein fraction was isolated (Pierce NE-PER), separated by SDS-PAGE, and transferred to nitrocellulose. The membrane was then probed with streptavidin-HRP (1 : 5000; Pierce) to bind biotinylated proteins, and the signal was detected by ECL (Pierce). Following detection of the biotinylated proteins from the nucleus, the (streptavidin) HRP on the membrane was inactivated by incubating the blot in PBS containing 3% H_2_O_2_ and 1% sodium azide. The same membrane was then reprobed with antibodies to CKAP4 (“anti-CLIMP-63”, diluted 1 : 1000, Alexis Biochemicals) and fibrillarin (a nuclear marker and loading control; Abcam; diluted 1 : 1000). (b) HeLa cells were treated with APF (20 nM) for 24 hours, which resulted in a significant increase in the abundance of CKAP4 in the nucleus compared to control samples. Treated cells were harvested and the nuclear and cytosolic fractions were isolated and separated by SDS-PAGE as described in Section 2. Protein expression was analyzed by Western Blotting with antibodies for *β*-tubulin (diluted 1 : 1000, Abcam; loading control for the nonnuclear fraction), CKAP4 (“anti-CLIMP-63”, diluted 1 : 1000, Alexis Biochemicals), and fibrillarin (diluted 1 : 1000, Abcam; loading control and specific marker for the nuclear fraction), and then with an HRP-conjugated anti-mouse secondary antibody (1 : 20000; ThermoFisher Scientific). The proteins were detected by ECL (Pierce) with multiple exposures to film. The integrated density of the bands on the film was measured using ImageJ. Exposure times were controlled to ensure that the signals on film were not saturated. (c) The nuclear/cytosolic ratio represents the relative distribution of CKAP4 in the nuclear versus cytosolic fractions extracted from cells treated with or without APF. CKAP4 abundance in the APF-treated and control samples were normalized for loading to *β*-tubulin for the nonnuclear fractions and to fibrillarin for the nuclear fractions. The nuclear/cytosolic ratio for CKAP4 in the APF and control samples was determined from these normalized values. The standard deviation describes the variability among the normalized, nuclear, and cytosolic ratios from three independent experiments. A two tailed, paired *t*-test of the two data arrays (plus APF and control) indicate that the difference between these ratios is significant (*P* = 0.01; *n* = 3). Cells treated with APF stop dividing, so the 10 cm dishes containing control and APF treated cells contained fewer cells (and protein) at the end of the experiment, normalizing the CKAP4 signals to loading controls corrected for this disparity. Fibrillarin is a well-characterized nuclear marker that is also known to localize to nucleoli. The data shown are representative of four independent experiments.

Previously, we observed by immunolocalization that CKAP4 is expressed on the surface of HeLa cells and became more abundant in the nucleus following APF exposure [6]. To confirm that CKAP4 translocates to the nucleus in response to APF, we used three different techniques—surface labeling, cellular fractionation, and immunocytochemistry. First, we surface-labeled HeLa cell proteins with sulfo-NHS biotin, a water soluble form of biotin that does not pass through the PM and binds only to primary amines of the extracellular portions of proteins. We then treated the cells with APF for three hours, isolated the nuclear protein fraction, and separated the proteins by SDS PAGE followed by transfer to nitrocellulose and detection with streptavidin-HRP. As demonstrated in Figure 2(a), biotinylated CKAP4 protein was detected in the nuclear fraction of APF-treated cells but not in the nuclear protein fraction isolated from untreated cells. This demonstrates that CKAP4 is present on the cell surface and that it is internalized and translocates to the nucleus in response to APF treatment.

**Figure 3:**
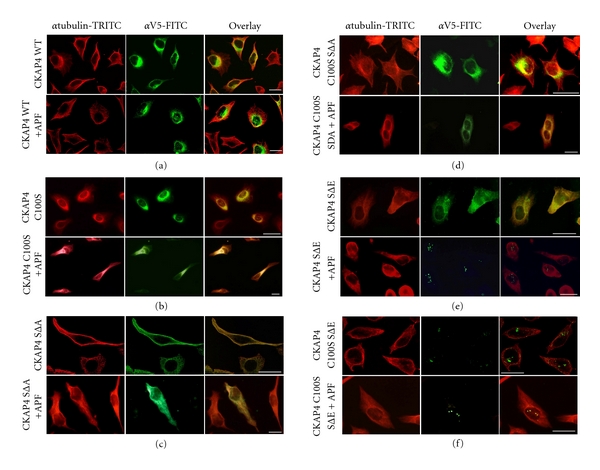
*CKAP4 phosphorylation and palmitoylation regulate CKAP4 trafficking*. CKAP4 mutants that mimic constitutive depalmitoylation and various states of serine [3, 17, 19] phosphorylation were generated to determine the effect of these two posttranslational modifications on the subcellular distribution of CKAP4 in response to APF (see Table 1). HeLa cells were transfected for 24–36 hours with the construct indicated to the left of each panel. Cells were serum starved for 6 hours and then treated with APF (20 nM) for 18–24 hours. Subsequently, the cells were fixed in 4% buffered paraformaldehyde, permeabilized with 0.1% Triton X-100, and immunostained for (1) *β*-tubulin (red; TRITC) and (2) V5 (green: FITC) (as described in Section 2) to distinguish the transfected, V5-epitope tagged WT (a) and mutant versions of CKAP4 from endogenous CKAP4 (b)–(d). Mutant versions of CKAP4 that cannot be palmitoylated (C100S) or phosphorylated (SΔA) do not translocate to the nucleus in response to APF. (e) Those that mimic phosphorylation (SΔE) translocate to the nucleus in response to APF. (f) CKAP4C100SSΔE, which is constitutively depalmitoylated and phosphomimicking, is expressed primarily in the nucleus. Images taken in each channel were superimposed to illustrate the distribution of mutant CKAP4 with respect to the cytoskeleton. The cells were imaged by epifluorescence or confocal microscopy at 60X and 63X, respectively, (Scale bars = 25 microns).

To determine whether there is a fractional change in nuclear abundance of CKAP4 in response to APF stimulation, we performed Western Blot analysis on nuclear and cytosolic protein fractions extracted from APF-treated and untreated cells. We found that exposure of HeLa cells to APF resulted in a ~4-fold increase in the relative abundance of CKAP4 in the nucleus (Figures 2(b) and 2(c)) confirming our earlier observations [6]. Lastly, we show by immunocytochemistry that transiently expressed, wild-type (WT) CKAP4 localizes to the nucleus after APF treatment (Figure 3(a)). Collectively, these data demonstrate that CKAP4 translocates to the nucleus of HeLa cells after APF treatment.

### 3.2. APF-Induced Nuclear Translocation of CKAP4 Is Regulated by Serine Phosphorylation

Previously we showed that DHHC2-mediated palmitoylation of CKAP4 on cysteine-100 (C100) is required for its nuclear localization in response to APF treatment [6]. Work by others has shown that phosphorylation of serines 3, 17, and 19 (S3, S17, and S19) also affects CKAP4 subcellular distribution, promoting disengagement from microtubules [7]. These findings suggest that APF binding may alter the palmitoylation and/or phosphorylation state of CKAP4, affecting its subcellular distribution and function within the cell. Consequently, we generated CKAP4 mutants that mimic constitutive depalmitoylation and various states of serine phosphorylation to examine the effect of APF on their subcellular distribution in HeLa cells using immunocytochemistry (Table 1). The CKAP4 mutations included C100 to serine (C100S) to block palmitoylation; mutation of S3, S17, and S19 to alanine (SΔA) to block phosphorylation; mutation of the same three serines to glutamic acid (SΔE) to mimic phosphorylation [7]. Each mutant was fused to a V5 epitope tag to immunologically distinguish it from endogenous CKAP4. Also, since phosphorylation regulates the association of CKAP4 with microtubules, we coimmunolabeled tubulin and CKAP4 to visualize differences in the relative localization of CKAP4 within the microtubule network. Table 1 provides a summary of the data.

As shown in Figure 3, we confirmed that palmitoylation was required for CKAP4 expression on the PM regardless of the state of phosphorylation (see Figures 3(b), 3(d), 3(f)). Importantly, when CKAP4 is not expressed on the PM, it does not respond to APF by a change in subcellular distribution (Figures 3(b), 3(d), and 3(f)). CKAP4C100S/SΔA expression was restricted to internal membranes and did not change in response to APF (Figure 3(d)). CKAP4SΔA was expressed throughout the cell, including the PM, and resembled that of WT CKAP4 (Figures 3(a) and 3(c)). Treatment of cells expressing CKAP4SΔA with APF did not cause translocation of the mutant form of CKAP4 into the nucleus, suggesting that CKAP4 must be phosphorylated on serine residues 3, 17, and 19, thus disengaged from the microtubule network, to translocate into the nucleus. CKAP4SΔE was also distributed throughout the cells including the PM, but in contrast to CKAP4SΔA, APF treatment caused translocation of CKAP4SΔE into the nucleus (Figure 3(e)). Surprisingly, CKAP4C100S/SΔE was expressed primarily if not exclusively in the nucleus (even in the absence of APF), and its localization did not change in response to APF treatment (Figure 3(f)). These data suggest that phosphorylation of CKAP4 on serine residues 3, 17, and 19 are required for its nuclear translocation in response to APF.

**Figure 4:**
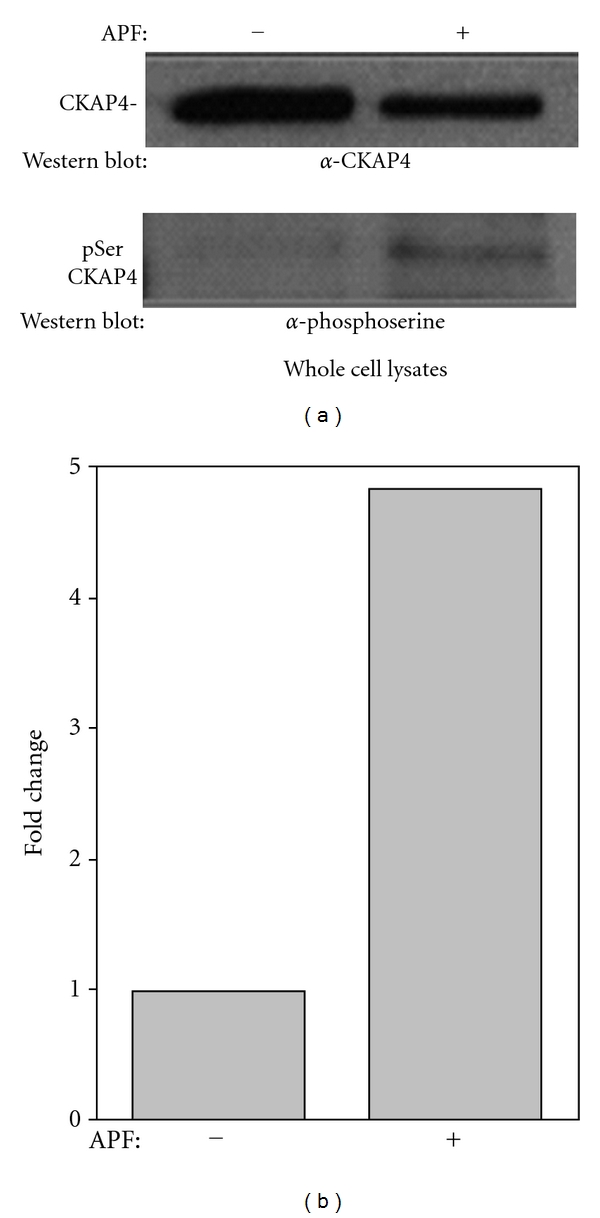
*APF induces serine phosphorylation of CKAP4*. (a) APF treatment induces a significant increase in serine phosphorylation of CKAP4 as demonstrated by immunoprecipitation of CKAP4 followed immunoblotting to detect phosphoserine. Whole cell lysates (500 *μ*g) from HeLa cells treated with 20 nM APF or serum-starved controls were immunoprecipitated with CKAP4 antibody (Alexis) overnight at 4°C. Samples were then bound to Protein A, washed (4X with RIPA/Empigen buffer), eluted (4X LDS sample buffer; Invitrogen), boiled at 95°C for 5 min and resolved on a 4–12% Bis-Tris gel and transferred to a nitrocellulose membrane under reducing conditions. Western Blot analysis for pSer detected phospho-serine using primary (Invitrogen and secondary antibodies (goat anti-rabbit HRP-labeled antibody; Pierce)) developed with Enhanced Chemiluminescence reagent (Pierce) and exposed to film. The membrane was stripped with Restore Stripping Buffer (Pierce) and reprobed for CKAP4 (Alexis) to normalize the phosphoserine signal to the amount of immunoprecipitated CKAP4. (b) Densitometric analysis of the immunoreactive bands was done using ImageJ, and the ratios of phosphorylated to nonphosphorylated CKAP4 were determined.

 To test the idea that APF treatment promotes serine phosphorylation of CKAP4, we used an antiphosphoserine antibody to measure any change in phosphorylation of endogenous CKAP4 from whole cell lysates in response to APF. Serine phosphorylation of CKAP4 immunoprecipitated from APF-treated HeLa cells was increased ~5.0-fold compared with CKAP4 immunoprecipitated from whole cell lysates of untreated cells (Figure 4), demonstrating that APF increases CKAP4 phosphorylation on serine residues.

### 3.3. Nuclear CKAP4 Is Phosphorylated following APF-Induced Translocation

**Figure 5:**
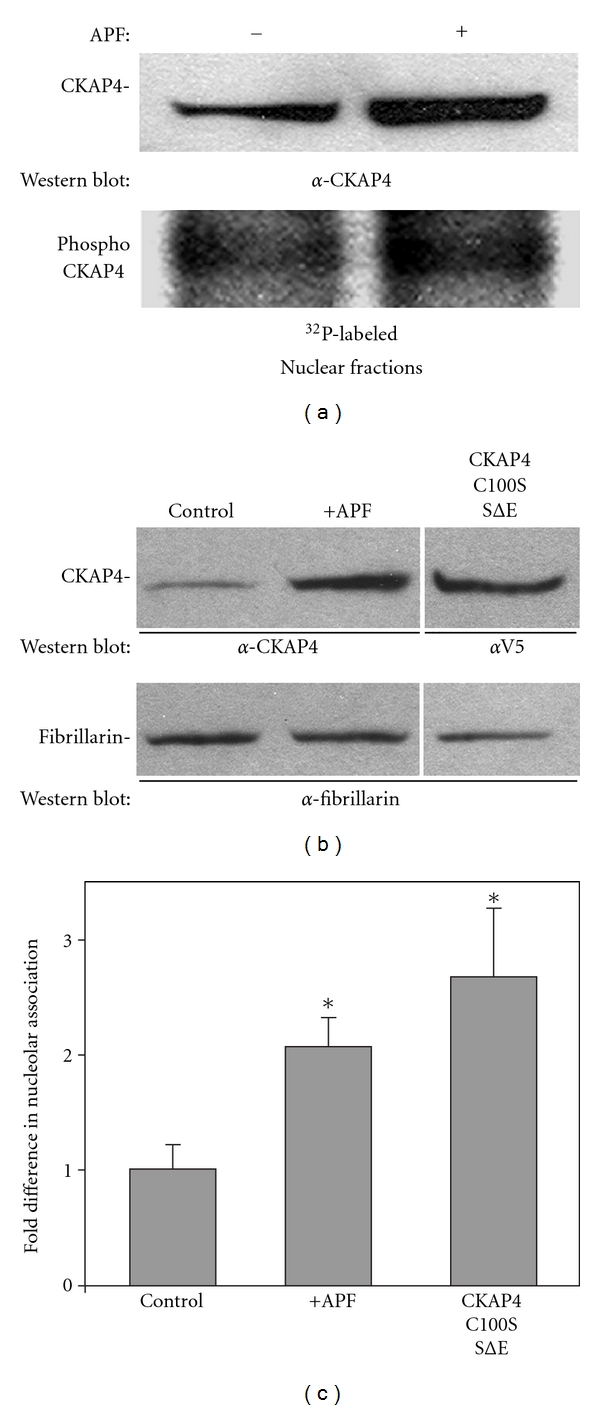
*Nuclear CKAP4 is phosphorylated following APF-induced translocation*. (a) CKAP4 in the nuclear fraction was phosphorylated to the same degree in APF-treated and control cells as demonstrated by metabolic labeling with *γ* ^32^P-ATP. The ratio of the CKAP4 phosphorylation signal over the corresponding CKAP4 Western Blot signal was approximately the same for both (1.00 versus 0.975; APF treated versus control). HeLa cells at ~80% confluence in 10 cm dishes were serum starved for 3 hours then 150 *μ*Ci *γ* ^32^P-ATP was added to each dish for 1 additional hour. Then the cells were either exposed to APF (20 nM; 24 hours) or left in serum-free medium (control) for 24 hours. At the end of 24 hours, the cells were harvested, washed three times in ice-cold PBS, and the nuclear and cytoplasmic fractions were isolated using the NE-PER (Pierce). Equal quantities of each fraction were separated by SDS-PAGE and transferred to nitrocellulose. The membrane was incubated overnight at 4°C in *α*-CKAP4 antibody (1 : 500) in TBST and 1% milk, washed and incubated for six hours at 4°C in a-mouse, HRP-conjugated secondary antibody (Pierce; 1 : 20,000) in TBST and 1% milk. CKAP4 bands were detected by enhanced chemiluminescence (ECL; Pierce; 20 second exposure; left panel). Following the Western Blot, the membrane was rinsed in 1% H_2_O_2_ for 1 minute to eliminate the chemiluminescent signal then wrapped in plastic wrap and exposed to film for 12 hours to detect the phosphorylated proteins. Densitometric analysis of the immunoreactive bands was done using ImageJ, and the ratios of phosphorylated to nonphosphorylated CKAP4 were determined. *APF increases the association of CKAP4 with nucleoli.* (b) and (c) Western Blot analysis shows that treatment of HeLa cells with APF (20 nM) increased the association of endogenous CKAP4 with the nucleolar fraction, and that the constitutively depalmitoylated and phosphomimicking CKAP4 mutant, CKAP4C100S/SΔE, associated with the nucleolar fraction to a greater extent than endogenous CKAP4 isolated from APF-treated cells. Nucleoli were isolated using a variation on the method published by Busch and coworkers [20] as described by the Angus Lamond lab (University of Dundee, UK). The nucleolar proteins were separated by SDS-PAGE and Western Blotted for CKAP4 (*α*-V5 in the case of CKAP4C100S/SΔE) and fibrillarin. The bands were detected on film by ECL and quantified using ImageJ. The CKAP4 signal in each lane of the Western Blot was normalized to the fibrillarin band in the same lane. The normalized value for CKAP4 from control cells was set to 1 and the other values were set relative to control. The values in the graph are means and SD. “*” indicates that the means of the values were significantly different than serum-starved control when evaluated using the students *t*-test (two-tailed. Serum starved versus APF treated *P* = 0.018; serum starved versus CKAP4C100S/SΔE *P* = 0.002; CKAP4C100S/SΔE versus APF treated, *P* = 0.008; *n* = 3 for each).

The observed increase in serine phosphorylation of endogenous CKAP4 and nuclear localization of the phosphomimicking CKAP4 mutants in response to APF suggested that endogenous CKAP4 should be phosphorylated if it is in the nucleus. To determine if nuclear CKAP4 is indeed phosphorylated, we metabolically labeled cells with *γ* ^32^P-ATP, exposed them to APF, and isolated the nuclear fraction. The level of phosphorylation was assessed by Western Blot after separation of proteins by SDS-PAGE. In agreement with previous experiments, the abundance of CKAP4 in the nucleus increased following exposure to APF (Figure 5(a), top panel). Nuclear CKAP4 was phosphorylated to an equal degree in both APF-stimulated and control cells (Figure 5(a), bottom panel). The ratio of the CKAP4 phosphorylation signal over the corresponding CKAP4 Western Blot signal was the same for both (1.00 versus 0.975; APF treated versus control) indicating that nuclear CKAP4 is phosphorylated.

In HeLa cells exposed to APF or cells transfected with CKAP4C100S/SΔE, we observed by immunolabeling that CKAP4 was not evenly distributed within the nucleus, but rather appeared to be concentrated on nucleoli and other unidentified subnuclear structures (data not shown). To determine whether CKAP4 was associated with nucleoli, we treated HeLa cells with APF for 24 hours and measured the abundance of CKAP4 in the nucleolar and nuclear fractions (nonnucleolar fraction of the nucleus) by Western Blot (Figure 5(b)). Our results demonstrate that APF induces an association of CKAP4 with nucleoli (Figures 5(b) and 5(c)). Immunolocalization of CKAP4C100S/SΔE suggested that it too associated with nucleoli (independent of APF treatment). To confirm this biochemically, we expressed CKAP4C100S/SΔE in HeLa cells, isolated the nucleolar fraction, and measured its association with nucleoli by Western Blot.  Figure 5 shows that CKAP4C100S/SΔE associates with nucleoli to a greater degree than endogenous CKAP4 in APF-treated cells. These data support the idea that CKAP4 becomes phosphorylated after binding to APF, and that phosphorylation enhances its nuclear translocation.

### 3.4. CKAP4C100S/SΔE Behaves as an APF Mimetic

**Figure 6:**
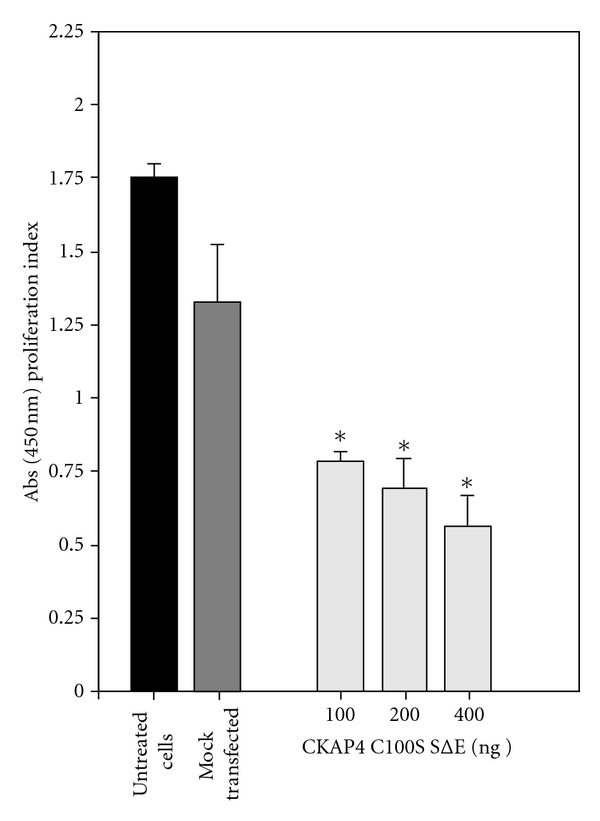
*CKAP4C100S/SΔE expression inhibits HeLa cell proliferation*. HeLa cells were mock transfected (Fugene; control) or transfected with 100, 200, or 400 ng of CKAP4C100S/SΔE DNA. After an additional 24 hours of growth, proliferation was measured using the WST-1 proliferation assay (Biovision) according to the manufacturer's protocol. Each data point is the mean and SD from 12 independent wells. Statistically significant differences in the rate of cellular proliferation were detected for HeLa cells transfected with 100, 200, and 400 ng of CKAP4C100S/SΔE DNA versus mock-transfected cells using the students *t*-test (*P < 0.001).

The similarity between the nuclear localization of endogenous CKAP4 following APF treatment and transiently expressed CKAP4 C100S/SΔE suggested that the mutant may mimic APF activity, such as inhibiting cellular proliferation. To determine if this hypothesis was correct, we transfected HeLa cells with varying quantities of CKAP4C100S/SΔE cDNA and measured their proliferation over the course of 48 hours.  Figure 6 shows that that transient expression of CKAP4C100S/SΔE significantly inhibits HeLa cell proliferation in a dose-dependent manner (versus mock transfected cells), a result that is characteristic of cells exposed to APF [6, 11, 21]. These results indicate that CKAP4C100S/SΔE may function as an APF mimetic and support the argument that endogenous CKAP4 is depalmitoylated and phosphorylated following APF binding.

### 3.5. Phosphorylated CKAP4 Binds to gDNA

**Figure 7:**
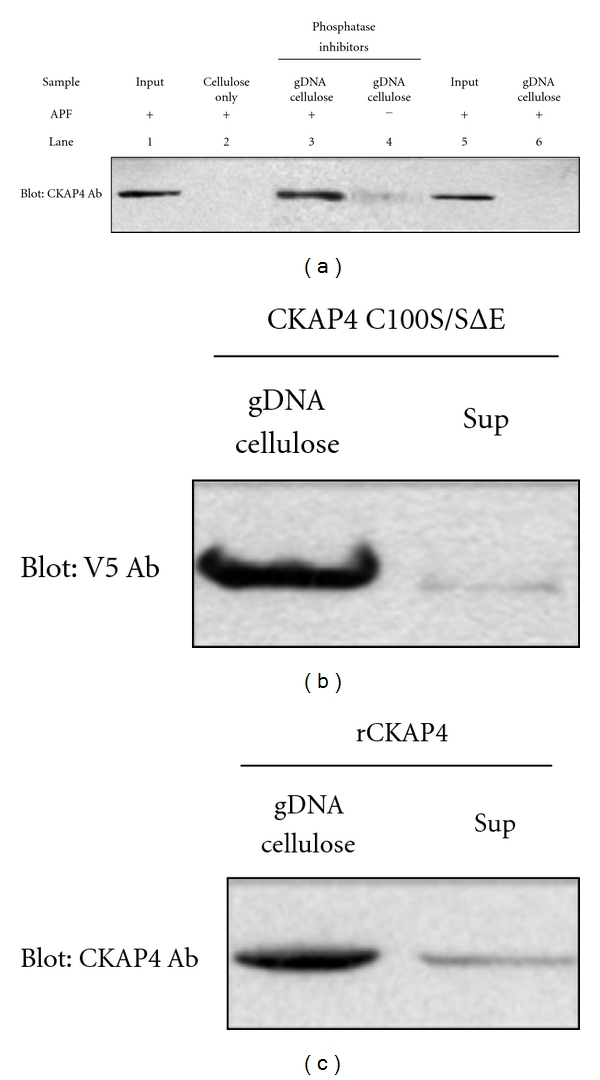
*CKAP4 binds directly to genomic DNA*. (a) CKAP4 binds gDNA in an APF-dependent manner (lane 3) when compared to mock-treated control (lane 4). The CKAP4-gDNA binding is phosphorylation dependent as CKAP4 isolated from cells treated without phosphatase inhibitors prior to APF treatment failed to bind gDNA (lane 6). As controls, we loaded APF treated nuclear lysates in lane 1 and phosphatase/APF treated lysates in lane 5. Lane 2 is a no gDNA cellulose control. (b) The ability of transiently transfected V5-tagged, CKAP4 C100S/SΔE to bind gDNA in the absence of APF treatment (c) and the ability of purified rCKAP4 (residues 126–501, which includes the bZIP-like DNA-binding domain) to bind gDNA were also assessed by comparing the amount of CKAP4 captured relative to what remained in the cell lysate after binding (Sup).

Because CKAP4 localizes to the nucleus/nucleolus in response to APF treatment and because its predicted structure includes a region homologous to bZIP transcription factors (Figure 1), we assessed whether CKAP4 could bind to DNA using genomic DNA (gDNA) affinity chromatography (GDAC)[22]. For this technique, nuclear lysates from APF- or mock-treated HeLa cells were incubated with gDNA-bound cellulose beads in the presence of nonspecific competitor (dI-dC), and proteins that bound DNA were isolated and separated by SDS-PAGE followed by Western Blot with an anti-CKAP4 antibody. As shown in Figure 7(a), CKAP4 binds gDNA in an APF-dependent manner (Lane 3) when compared to mock-treated control (Lane 4). The CKAP4-gDNA binding is phosphorylation dependent as CKAP4 isolated from cells that were treated without phosphatase inhibitors prior to APF treatment failed to bind gDNA (Lane 6). Furthermore, in nuclear lysates from cells transfected with the V5-tagged, phosphomimicking CKAP4 mutant (CKAP4 C100S/SΔE), we observed enhanced binding to gDNA (Figure 7(b)). In Figure 7(c), we show that purified rCKAP4 (C-terminal residues 126–501, which includes the bZIP-like DNA-binding domain) also binds gDNA. Collectively, these data suggest that APF induces phosphorylation of CKAP4 and that phosphorylation is required for CKAP4 to bind gDNA.

## 4. Discussion

CKAP4 has an established role in maintaining the structure of the ER relative to the cytoskeleton by binding to microtubules—a function which is dependent on three N-terminal serine residues (S3, S17, and S19) that when phosphorylated, cause CKAP4 to disengage from microtubules (see Figure 1) [3, 7]. Expression of mutant versions of CKAP4 that do not bind microtubules profoundly changes ER morphology [2]. More recently, our knowledge of CKAP4 function has expanded to include it being a cell surface receptor for APF [10], surfactant protein A (SPA) [23], and tissue plasminogen activator (tPA) [24]. In a previous study, we showed that palmitoylation of CKAP4 by DHHC2 is required for its localization on the PM and for mediating APF-induced signaling events, including inhibition of cellular proliferation and changes in gene/protein expression [6]. However, prior to the data presented here, there was nothing known about the mechanism for APF signal transduction by CKAP4.

Previously, we observed by immunocytochemistry that exposing cells to APF resulted in an increased abundance of CKAP4 in the nucleus [6]. These data suggested that APF binding to CKAP4 on the cell surface induced its translocation into the nucleus. In this study, we used cell-surface labeling with Sulfo-NHS-biotin (which reacts only with extracellular primary amines but does not bind to proteins inside of the cell during the labeling process) to monitor CKAP4 localization in response to APF binding. Our results showed that biotinylated CKAP4 from the cell surface is abundant in the nuclear fraction of HeLa cells treated with APF, but not in untreated cells. Detection of a relatively small amount of nonbiotinylated CKAP4 in the nucleus of untreated cells in this experiment is consistent with data from other experiments, indicating that CKAP4 may have some inherent nuclear function not related to APF-induced signal transduction, moreover, it suggests that in order to block proliferation, CKAP4 in the nucleus must have bound to APF at some point.

Using a series of mutants that mimic combined states of palmitoylation and phosphorylation, we determined that APF promotes serine phosphorylation of CKAP4; moreover, that phosphorylation of serines 3, 17, and 19 is required for APF-induced nuclear translocation of CKAP4, as mutation of these residues to alanine (SΔA) inhibited this process. Additionally, mutation of these serine residues to glutamic acid to create a phosphomimicking form of CKAP4 (CKAP4SΔE) promoted the nuclear translocation of CKAP4 in response to APF. Interestingly, a phosphomimicking form of CKAP4 that was also constitutively depalmitoylated localized in the nucleus without APF stimulation. These data suggest that when CKAP4 is bound by APF on the PM, it becomes depalmitoylated and phosphorylated and translocates into the nucleus. In direct support of the idea that CKAP4 must be phosphorylated to enter the nucleus, we show by metabolic labeling with *γ* ^32^P-ATP that nuclear CKAP4 is phosphorylated. Despite using multiple methods, we did not observe palmitoylated CKAP4 in the nucleus. The mechanism by which CKAP4 or any other palmitoyl-protein becomes depalmitoylated is a matter of great interest. However, there is very little known about how depalmitoylation is regulated. Serines 3 and 19 reside within a consensus site for PKC and serine 17 within a CKII site [7]; however, whether these kinases are responsible for phosphorylation of these serines remains to be determined.

CKAP4 has a ~106-amino acid cytosolic tail, a single TM domain, and a large, extracellular/ER-luminal domain consisting of 474 amino acids (see Figure 1). Much of the extracellular domain, including the portion required for oligomerization, is predicted to fold into a coiled-coil conformation, with an amphipathic alpha helix rich in basic residues comprising the C-terminal (~80) residues [3]. These structures are among the most common and well-characterized domains involved in protein-protein interactions. Moreover, amino acid residues 468–602 are homologous to the DNA-binding domain of bZIP transcription factors. Many details about the predicted structure of CKAP4, its nonrandom distribution in the nucleus, and enhanced association with nucleoli (see Figure 5), suggest that it might bind to DNA. Genomic DNA affinity chromatography is a straight-forward method to determine if a protein binds to DNA without *a priori* knowledge of the exact binding sites [25]. Using GDAC, we demonstrated that CKAP4 isolated from APF-treated HeLa cells bound genomic DNA (gDNA). Furthermore, our results show that inclusion of phosphatase inhibitors was required for endogenous CKAP4 to bind to gDNA, but not for binding of the purified CKAP4 C-terminal fragment to gDNA. This indicates that the C-terminal fragment alone, separated from the phosphoserines near the N-terminus, is free to reside in a conformational state that favors gDNA binding—the same conformation that exists in the endogenous protein when phosphorylated. Future experiments will be carried out to identify the specific DNA sequences to which CKAP4 binds.

CKAP4 is 63 kDa, homo-oligomeric, and is embedded in membranes; these are not typical, physical properties of proteins that enter the nucleus by diffusion. Generally, proteins that diffuse into the nucleus are cytosolic and have a molecular weight less than 40 kDa. Proteins too large to diffuse through the nuclear pore translocate with the assistance of a shuttle protein (or karyopherin) that is physically bound to a nuclear localization signal (NLS) sequence of the imported protein. CKAP4 contains a glycine-rich domain that may act as an NLS. Similar domains found in several families of proteins are known to mediate nuclear import [22, 26]. Ongoing work indicates that this domain of CKAP4 is sufficient to drive nuclear localization of a nonrelated protein (data not shown).

Because of its size and TM domain, it would seem unlikely that CKAP4 would have a nuclear function. However, there is precedent for large, TM-domain proteins entering the nucleus. The EGF (epidermal growth factor) receptor family are glycosylated, single-pass TM domain proteins of ~140 kDa that also translocate, unproteolyzed, from the plasma membrane into the nucleus after ligand binding [27]. Within the nucleus, they regulate transcription and participate, enzymatically, in signal transduction pathways. The idea that these proteins translocated to and had functional activity in the nucleus, beyond their tyrosine kinase activity at the plasma membrane, was controversial; however, more recently, the mechanistic details about how they make their way into the nucleus and how they function within the nucleus are becoming clearer (reviewed in [27]).

In summary, the data presented here provide new insight into the mechanism by which CKAP4 mediates APF signal transduction. At the same time, we have added to a small but growing body of literature demonstrating that a receptor in the plasma membrane can translocate into the nucleus after binding ligand. Finally, our data demonstrate that CKAP4C100S/SΔE has APF-like activity as it localizes to the nucleus and inhibits proliferation without binding to APF. This finding suggests that CKAP4C100S/SΔE could be used to treat hyperproliferative diseases such as cancer. APF itself has been proposed as an anticancer therapy [28].

## Figures and Tables

**Table 1:** *Mutant constructs used to study CKAP4 phosphorylation and palmitoylation*. CKAP4 mutants that mimic constitutive depalmitoylation and various states of serine (S3, S17 and S19) phosphorylation were generated to determine their effect on the subcellular distribution of CKAP4 in response to APF. CKAP4SΔE translocated from the PM to the nucleus in response to APF; CKAP4SΔA localized to the PM but did not translocate to the nucleus in response to APF; none of the C100S mutants were expressed on the PM; CKAP4C100S/SΔE was expressed in the nucleus. The results indicate that CKAP4 must be palmitoylated for PM expression and depalmitoylated and phosphorylated for translocation to the nucleus. A summary of these results are provided in the table.

Name	Mutations	Comments posttranslational modifications	Resting distribution	Distribution after APF
wild-type (WT) CKAP4	none	expressed or endogenous	PM, ER	> nucleus
CKAP4 C100S	C100S	constitutively depalmitoylated	ER	= ER
CKAP4 SΔA	S3A, S17A, S19A	constitutively dephosphorylated	PM, ER	= PM, ER
CKAP4 C100S/SΔA	C100S, S3A, S17A, S19A	constitutively depalmitoylated, constitutively dephosphorylated	ER	ER
CKAP4 SΔE	S3E, S17E, S19E	mimics phosphorylation	PM, ER	> nucleus
CKAP4 C100S/SΔE	C100S, S3E, S17E, S19E	constitutively depalmitoylated, mimics phosphorylation	Nucleus	= nucleus

## Abbreviations

APF: Antiproliferative factor
CKAP4: Cytoskeleton-associated protein 4/p63
DHHC: Asp-His-His-Cys
DMEM: Dulbecco's modified Eagle's medium
ER: Endoplasmic reticulum
FBS: Fetal bovine serum
FITC: Fluorescein isothiocyanate
GDAC: Genomic DNA affinity chromatography
gDNA: Genomic DNA
IC: Interstitial cystitis
LZ: Leucine zipper
mAb: Monoclonal antibody
MT: Microtubule
NGS: Normal goat serum
NLS: Nuclear localization signal
PAGE: Polyacrylamide gel electrophoresis
PAT: Palmitoyl acyltransferase
PBS: Phosphate-buffered saline
PM: Plasma membrane
SDS: Sodium dodecyl sulfate
SP-A: Surfactant protein-A
TM: Transmembrane
tPA: Tissue plasminogen activator
TRITC: Tetramethyl rhodamine isothiocyanate.
